# Chemokine-derived oncolytic peptide induces immunogenic cancer cell death and significantly suppresses tumor growth

**DOI:** 10.21203/rs.3.rs-3335225/v1

**Published:** 2023-10-03

**Authors:** Natsuki Furukawa, Wendy Yang, Alex Chao, Akash Patil, Adam Mirando, Niranjan Pandey, Aleksander Popel

**Affiliations:** Johns Hopkins University School of Medicine; Johns Hopkins University School of Medicine; Johns Hopkins University School of Medicine; Johns Hopkins University School of Medicine; Johns Hopkins University School of Medicine; Johns Hopkins University School of Medicine; Johns Hopkins University School of Medicine

## Abstract

Chemokinostatin-1 (CKS1) is a 24-mer peptide originally discovered as an anti-angiogenic peptide derived from the CXCL1 chemokine. Here, we demonstrate that CKS1 acts not only as an anti-angiogenic peptide but also as an oncolytic peptide due to its structural and physical properties. CKS1 induced both necrotic and apoptotic cell death specifically in cancer cells while showing minimal toxicity in non-cancerous cells. Mechanistically, CKS1 disrupted the cell membrane of cancer cells quickly after treatment and activated the apoptotic pathway at later time points. Furthermore, immunogenic molecules were released from CKS1 treated cells, indicating that CKS1 induces immunogenic cell death. CKS1 effectively suppressed tumor growth *in vivo*. Collectively, these data demonstrate that CKS1 is a unique peptide that functions both as an anti-angiogenic peptide and as an oncolytic peptide and has a therapeutic potential to treat cancer.

## Introduction

Currently, most drugs used for cancer treatment are small molecules or antibodies [1]. In general, small molecules show low affinity and specificity towards their targets which causes undesired side effects. Although therapeutic antibodies are well suited to interfere with protein-protein interactions, the poor tissue penetration due to their large size is a prominent drawback. High production costs also limit their availability to patients [2]. Peptides possess unique properties that can overcome the disadvantages of these therapies [3, 4]. Similar to antibodies, peptides can bind to their target protein with high affinity and high specificity. Moreover, peptides are small and can penetrate tissues better than antibodies. Peptides are synthesized by chemical reactions which makes the production costs significantly lower than antibodies that require the usage of biological materials and protein purification. Therefore, peptide therapeutics have the potential to be effective and low-cost cancer treatments.

Chemokinostatin-1 (CKS1) is a 24-mer peptide originally discovered as an anti-angiogenic peptide. Karagiannis and Popel developed a computational method to identify anti-angiogenic sequences contained in the human proteome and discovered common sequences conserved within protein domains contained in angiogenesis-related proteins [5]. CKS1 is derived from a sequence shared in the CXC chemokine family. The anti-angiogenic property of CKS1 was verified experimentally [6], and its efficacy in suppressing tumor growth was demonstrated in triple-negative breast cancer (TNBC) [7] and glioma [8] models.

Oncolytic peptides selectively induce cell death in cancer cells. They typically have an amphipathic structure and a net positive charge [9]. Oncolytic peptides interact with the negatively charged molecules on the cell membrane, such as phosphatidylserine and glycosylated proteins, via electrostatic interactions. Once the concentration of the peptide on the surface of the cancer cells exceeds a threshold, the peptides undergo a conformational change to disrupt the cell membrane and eventually induce cell death. Three major models have been proposed to explain the cell penetration step [10]. In the barrel pore model, the peptides span through the membrane to form a pore. The toroidal pore model proposes that the peptides interact with the lipids in the cell membrane to form a pore composed of peptides and lipids. In the carpet model, the peptides cover the surface of the cell membrane and carve out the lipids by forming micelle-like particles.

The specificity of oncolytic peptides for cancer cells over non-cancer cells is explained by the differences in the components of the cell membrane. In healthy cells, flippases and floppases function to maintain the asymmetric distribution of phospholipids. Neutral phospholipids such as sphingomyelin and phosphatidylcholine are mostly located in the outer layer of the cell membrane, while negatively charged phospholipids such as phosphatidylserine and phosphatidylinositol are sequestered in the inner layer of the cell membrane. Due to this asymmetric distribution of phospholipids, the cell surface of healthy cells is neutral [11]. However, the machinery to maintain the asymmetric distribution is frequently impaired in cancer cells, leading to the exposure of negatively charged phospholipids. Accordingly, the cell surface of cancer cells is commonly more negatively charged compared to healthy cells [12]. High level of lactate production due to the propensity of cancer cells to upregulate glycolysis also contributes to the negative charge of cancer cell membrane [13].

Since oncolytic peptides are positively charged, they preferentially bind to the cell surface of cancer cells [14]. As a proof of concept, Iwasaki et al. demonstrated that cell lines with higher expression of phosphatidylserine were more sensitive to oncolytic peptides [15]. Moreover, the fluidity of the cell membrane also determines the sensitivity to oncolytic peptides. Cholesterol in the cell membrane decreases the fluidity of the cell membrane and makes it difficult for the oncolytic peptides to insert into the lipid bilayer [14]. Some cancer cell types exhibit higher membrane fluidity due to the decreased amount of cholesterol in the membrane, which leads to higher sensitivity to oncolytic peptides [12].

In addition to the disruption of the cell membrane, disruption of the organelles may also be important for the activity of oncolytic peptides. LTX-315, a 9-mer oncolytic peptide, has been shown to accumulate in the mitochondria and cause mitochondrial outer membrane permeabilization [16, 17]. Treatment with LTX-315 leads to the release of cytochrome-c and generation of reactive oxygen species (ROS). Importantly, inhibition of mitochondrial activity with high concentration of carbonyl cyanide mchlorophenyl hydrazone (CCCP), an inhibitor of oxidative phosphorylation, decreased the cytotoxicity of LTX-315. Another oncolytic peptide, LTX-401, accumulates in the Golgi membranes and destroys the Golgi apparatus [18]. Although the molecular mechanism of how the disruption of Golgi apparatus leads to cell death remains unclear, inhibition of protein transport into the Golgi apparatus using Brefeldin A led to decreased cytotoxic activity of LTX-401. Despite LTX-315 and LTX-401 both being amphipathic peptides with membrane-disrupting capability, LTX-315 accumulates in the mitochondria but not in the Golgi apparatus, whereas LTX-401 localizes mostly in the Golgi apparatus and the cytosol, and not in the mitochondria [18]. Wodlej et al. compared the localization of R-DIM-P-LF11–322, which is an oncolytic peptide that interacts specifically with cancer cells, and DIM-LF11–318, which is an oncolytic peptide that also induces cell death of non-cancerous cells [19]. R-DIM-P-LF11–322 accumulated in the Golgi apparatus and induced swelling of the mitochondria and eventually apoptosis. On the other hand, the type of cell death caused by DIM-LF11–318 was mainly necrosis caused by the rupturing of the cell membrane. These data indicate that each oncolytic peptide exhibits a unique subcellular localization which may affect the mechanism of cell death caused by each peptide.

Cell death caused by oncolytic peptides has been reported to induce the release of immunogenic molecules from the cells [20–22]. Oncolytic peptides contribute to the treatment of cancer not only by killing cancer cells but also by stimulating anti-tumoral immunity. LTX-315 has been shown to synergize with anti-CTLA4 antibodies in a mouse sarcoma model [23]. In a small clinical trial, LTX-315 treatment led to increased expression of immune-related genes, increased T-cell clone expansion in blood, and increased infiltration of immune cells into tumors in melanoma, sarcoma, and breast cancer [24]. Currently, several clinical trials evaluating the efficacy of LTX-315 monotherapy and combinations of LTX-315 with immunotherapies are being conducted.

Although CKS1 was originally identified as an anti-angiogenic peptide, its predicted structural properties indicated potential oncolytic activity. Here we use several biochemical and imaging assays to confirm cancer-specific membrane disruption and subsequent apoptosis while minimally impacting normal cells. This mechanism of cell death was also found to release the immunogenic molecules, suggesting possible synergy with immune modulation. Finally, anti-tumor activity was demonstrated in two mouse models. Together with the previously discovered anti-angiogenic properties, the oncolytic activities uncovered in these studies emphasize the potential of CKS1 as a promising cancer therapeutic.

## Results

### CKS1 has a suitable structure as an oncolytic peptide

**Figure 1:**
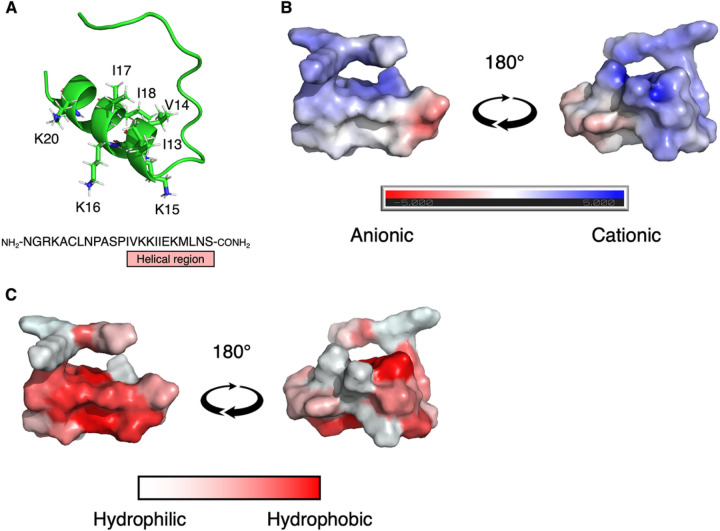
The structure and physical properties of CKS1. (**A**) The structure of CKS1 predicted by AlphaFold 2. (**B**) The electrostatic potential of the surface of CKS1 was predicted by APBS Electrostatics plugin in Pymol. Blue regions are the positively charged regions and the red regions are the negatively charged regions. (**C**) The hydrophobic regions of CKS1 are visualized as red, and the hydrophilic regions are visualized as white.

CKS1 (NGRKACLNPASPIVKKIIEKMLNS) is predicted to contain an unstructured N-terminal region and a Cterminal α-helix (Fig. 1A). Importantly, CKS1 contains several basic amino acids that make the net charge positive (Fig. 1B). The charged amino acids are concentrated on one side of the helix, and the other side of the helix is formed by hydrophobic amino acids (Fig. 1C). The cationic amphipathic helical structure of CKS1 is consistent with that of oncolytic peptides. Furthermore, using ACPred, a support vector machinebased machine learning model, we predicted that CKS1 would be an oncolytic peptide [26]. These findings indicated that CKS1 could function as an oncolytic peptide.

### CKS1 induces rapid cell death of cancer cells

**Figure 2:**
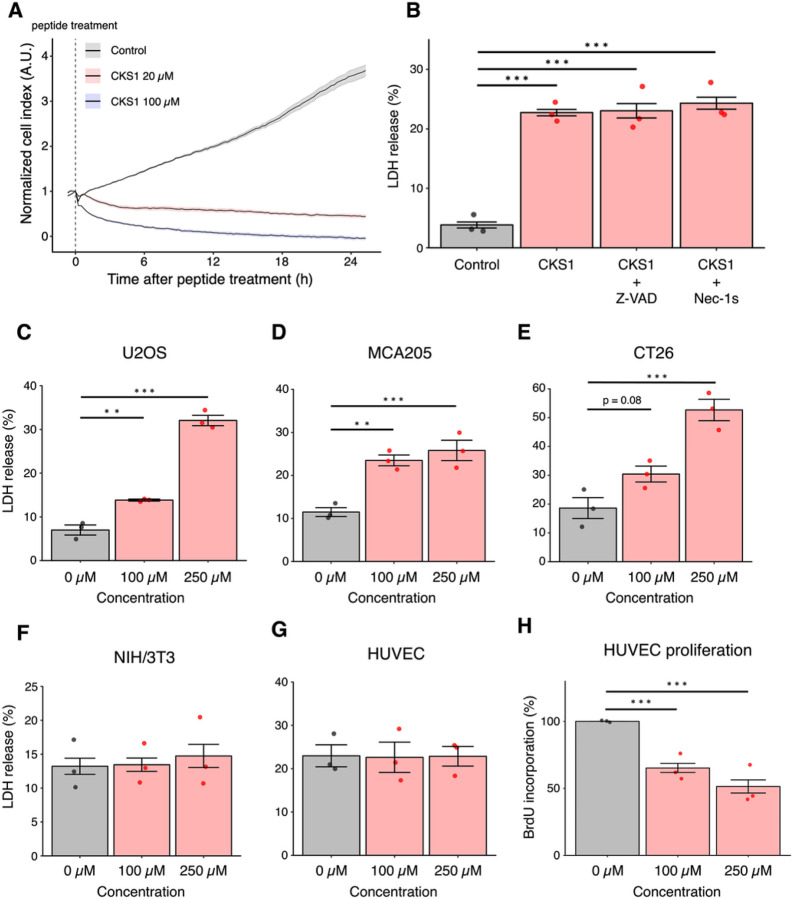
CKS1 induces rapid cell death in multiple cancer cell lines but not in non-cancerous cell lines. (**A**) Cell viability of 4T1 cells after CKS1 treatment was measured by real-time cell analyzer (RTCA). The vertical, dotted line indicates the time of peptide addition, and solid lines and the shaded areas show the mean and the standard error of the technical replicates, respectively. Representative of N = 3. (**B**) 4T1 cells were pre-treated with 50 μM pan-caspase inhibitor Z-VAD for 30 minutes or 50 μM RIPK1 inhibitor Nec-1s for 30 minutes. After the pre-treatment, 4T1 cells were treated with 100 μM CKS1 for 6 hours. The release of LDH in the supernatant was measured by the CyQUANT LDH cytotoxicity assay and was normalized to the amount of LDH released from cells treated with lysis buffer. Means ± SEM, N = 3, Dunnett’s test (***p < 0.005). (**C - G**) U2OS cells, MCA205 cells, CT26 cells, NIH/3T3 fibroblasts, and HUVECs were treated with the indicated concentration of CKS1 for 6 hours. The release of LDH in the supernatant was measured and normalized as described in (B). Means ± SEM, N = 3, Dunnett’s test (**p < 0.01, ***p < 0.005). (**H**) HUVECs were treated with CKS1 and the incorporation of BrdU was measured. Means ± SEM, N = 3, Dunnett’s test (***p < 0.005).

To determine whether CKS1 functions as an oncolytic peptide, we monitored the cell viability of 4T1 murine TNBC cells after CKS1 treatment using xCELLigence Real-Time Cell Analyzer (Agilent). In this method, the binding of cells to gold electrodes is detected as electrical impedance and displayed as arbitrary units of cell index in real-time. Cell death, as suggested by a reduction in cell index, was observed within 1 hour of treatment and was dose-dependent (Fig. 2A). We further quantified the release of lactate dehydrogenase (LDH) in the culture media after CKS1 treatment. LDH is a cytosolic enzyme that is released outside the cells when the cell membrane is damaged. Cells treated with CKS1 released significant amounts of LDH showing that CKS1 induces damage in the cell membrane of 4T1 cells. Next, we attempted to clarify whether the cell membrane damage was induced by intrinsic cellular machinery or by physical damage caused by the peptides. Pretreatment with the pan-caspase inhibitor Z-VAD or RIPK1 inhibitor necrostatin-1 stable (Nec-1s) did not inhibit the release of LDH, indicating that the release of LDH by CKS1 does not require these programmed cell death pathways (Fig. 2B). The cytotoxicity of CKS1 was not limited to 4T1 cells, and CKS1 was found to be effective in several murine and human cancer cell lines: human osteosarcoma U2OS cells, murine fibrosarcoma MCA205 cells, and murine colon carcinoma CT26 cells (Fig. 2C – E). Importantly, CKS1 did not induce cell death of NIH/3T3 normal fibroblasts and human umbilical vein endothelial cells (HUVECs) (Fig. 2F, 2G). Although CKS1 did not induce LDH release, CKS1 significantly attenuated cell proliferation of HUVECs as demonstrated by the inhibition of BrdU incorporation (Fig. 2H). These results indicate that the reported anti-angiogenic property is separate from the cell death-inducing capacity of CKS1.

### CKS1 rapidly disrupts the cell membrane and induces apoptosis at later time points

**Figure 3:**
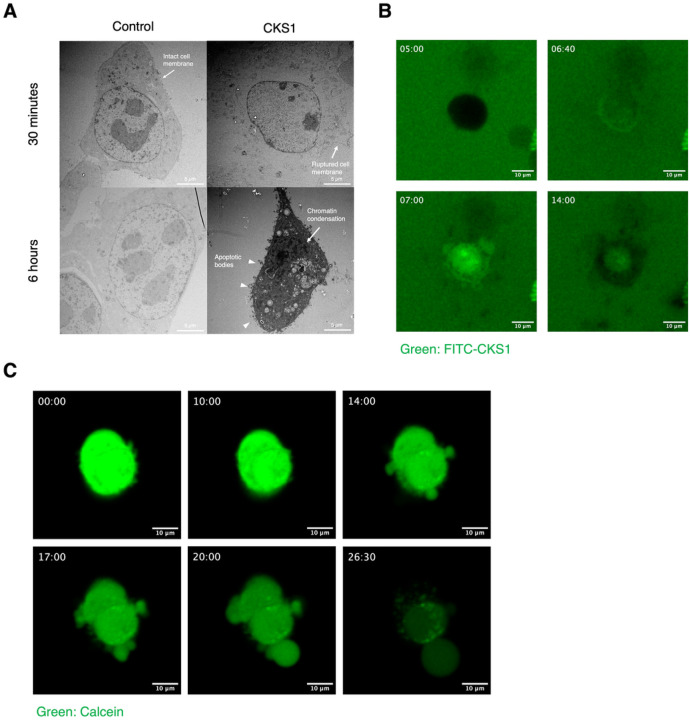
CKS1 rapidly disrupts the cell membrane and induces apoptosis at later time points. (**A**) 4T1 cells were treated with water or 100 μM CKS1 for the indicated time. The detailed structure of the cell surface was observed using transmission electron microscopy. 4T1 cells had intact cell membrane in the control condition, but the cell membrane was disrupted when they were treated with CKS1 for 30 minutes. When cells were treated with CKS1 for 6 hours, 4T1 cells exhibited an apoptotic morphology. Overall shrinkage of the cell, collapse of the nucleus, chromatin condensation (arrow), and apoptotic blebbing (triangle) indicate that the cells underwent apoptosis. (**B**) 4T1 cells were treated with 100 μM FITC-CKS1 (Green) and monitored by fluorescence live cell imaging. The timestamp indicates the minutes after cells were treated with the peptide. Representative of N = 3. (**C**) 4T1 cells were stained with calcein-AM (Green) and were treated with 100 μM CKS1. The timestamp indicates the minutes after cells were treated with the peptides.

To identify the mechanism of cell death caused by CKS1, we observed the morphology of the cells after CKS1 treatment using electron microscopy. The cell membrane of cells treated with CKS1 for 30 minutes was ruptured. The nuclear membrane was not ruptured by 30 minutes-treatment of CKS1 (Fig. 3A). When the cells were treated with CKS1 for 6 hours, we observed cells with an apoptotic morphology. The volume of the cells shrank, the nucleus collapsed, the chromatin condensed, and apoptotic blebbing was observed. These data indicate that CKS1 induces caspase-independent necrotic cell death by disrupting the cell membrane at early time points and induces apoptosis at later time points after CKS1 treatment.

To observe the interactions between the peptide and 4T1 cells, we produced CKS1 tagged with fluorescein isothiocyanate (FITC) and observed its movement after adding it to the cells (Fig. 3B, **Supplemental Video 1, 2**). FITC-CKS1 first accumulated on the cell surface which is seen as a ring around the cell. Next, the peptides entered the cells. To further clarify the events leading to cell death, we stained the cytosol of 4T1 cells using calcein-AM and treated them with CKS1 (Fig. 3C, **Supplemental Video 3, 4**). After CKS1 treatment, there was an influx of the surrounding media into the cells. The influx caused swelling of the cells and blebbing to occur. The blebbing eventually bursts, leading to the release of cytosolic components into the extracellular space.

### CKS1 diminishes mitochondrial membrane potential and activates the apoptotic pathway

**Figure 4:**
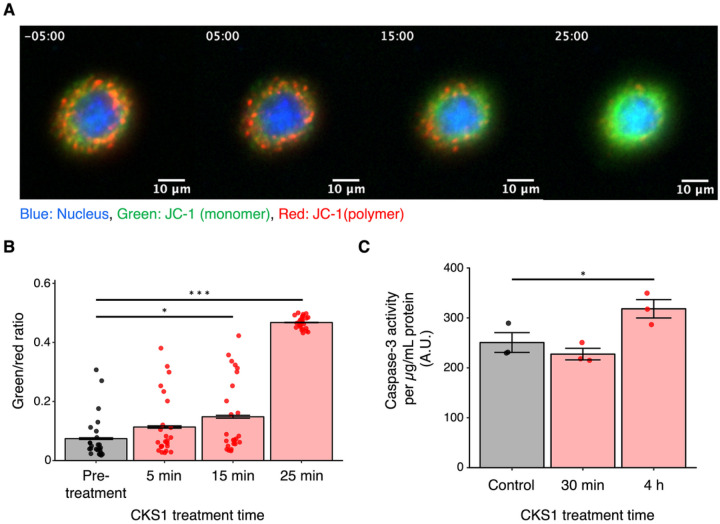
CKS1 diminishes mitochondrial membrane potential and activates the apoptotic pathway. (**A**) Fluorescence live cell imaging of 4T1 cells stained with JC-1 and treated with 100 μM CKS1. The timestamp indicates the minutes after CKS1 treatment (negative value indicates pre-treatment). Green indicates the monomer form of JC-1, and red indicates the polymer form of JC-1. (**B**) 25 cells were selected from the video of figure A (**supplemental video 5**) and the ratio between the green intensity and the red intensity was calculated. Means ± SEM, representative of N = 3 independent experiments, Dunnett’s test (*p < 0.05, ***p < 0.005). (**C**) 4T1 cells were treated with 100 μM CKS1 for the indicated time. The activity of caspase-3 was quantified by caspase-3 assay. The measured fluorescence was divided by the protein concentration in the cell lysate. Means ± SEM, N = 3, Dunnett’s test (*p < 0.05).

Since we observed an apoptotic morphology of 4T1 cells treated with CKS1, we hypothesized that CKS1 may disrupt the function of the mitochondria and activate the apoptotic pathway. To test this hypothesis, we stained 4T1 cells with JC-1. JC-1 is a cationic dye that accumulates in the mitochondria. In intact mitochondria, when the mitochondrial membrane potential is high, JC-1 forms aggregates that emit red fluorescence. When the mitochondrial membrane potential is low, JC-1 exists as monomers that emit green fluorescence. Therefore, the ratio of green and red fluorescence emitted by the two forms of JC-1 functions as an indicator of the mitochondrial membrane potential. We observed a rapid switch of JC-1 emitted fluorescence from red to green when 4T1 cells were treated with CKS1 (Fig. 4A, B, **Supplemental Video 5, 6**). This indicates that CKS1 rapidly diminishes the mitochondrial membrane potential in 4T1 cells. Furthermore, the activity of caspase-3 increased after CKS1 treatment (Fig. 4C). These data suggest that in addition to cell death caused by the rupturing of the cell membrane, disruption of the mitochondrial activity and the subsequent activation of the apoptotic pathway may contribute to the cell death caused by CKS1.

### The helical region of CKS1 drives the oncolytic activity

**Figure 5:**
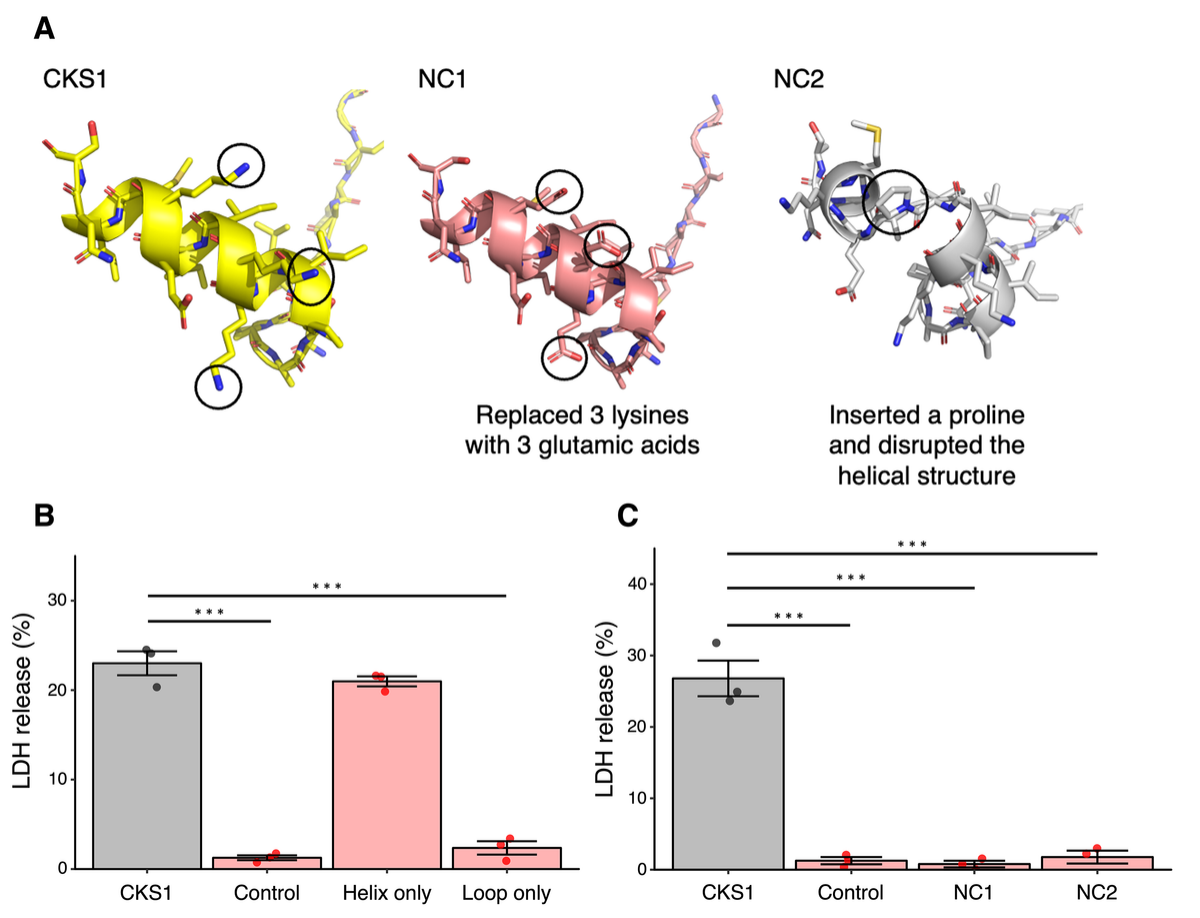
The cationic helical structure of CKS1 is important for its activity. **(A)** The structure of CKS1, NC1 and NC2. (**B, C**) The release of LDH in the supernatant was measured after treating 4T1 cells with 100 μM of the indicated peptides for 6 hours. Data were normalized to cells treated with lysis buffer. Means ± SEM, N = 3, Dunnett’s test (***p < 0.005).

Our motivation to test the oncolytic activity of CKS1 was the observation that CKS1 possesses the characteristic amphipathic, positively charged α-helix. To examine whether these features are responsible for the oncolytic activity, we produced a peptide that only contains the C-terminal helical region (IVKKIIEKMLNS) and a peptide that contains the N-terminal loop region (NGRKACLNPASP). Furthermore, we produced two mutant peptides, NC1 and NC2 (Fig. 5A). In NC1, the three lysines that form the hydrophilic side of CKS1 were replaced with glutamic acids. Since lysines are positively charged and glutamic acids are negatively charged, these mutations make the peptide anionic rather than cationic. In NC2, a single proline was inserted in the middle of the α-helix of CKS1. Since prolines destabilize α-helices, the helical structure of CKS1 would be expected to be disrupted in NC2. As expected, the peptide with just the helical region of CKS1 induced LDH release from 4T1 cells, while the peptide with only the loop region of CKS1 did not (Fig. 5B). Although the loop region was not directly involved in the oncolytic activity, it may enhance the oncolytic activity of CKS1. For example, the peptide with only the helical region was poorly soluble in water, while CKS1 showed high solubility in water (data not shown). NC1 and NC2 did not show oncolytic activity, demonstrating that the cationic property and the helical property are both essential for the oncolytic activity of CKS1 (Fig. 5C).

### CKS1 induces immunogenic cell death

**Figure 6:**
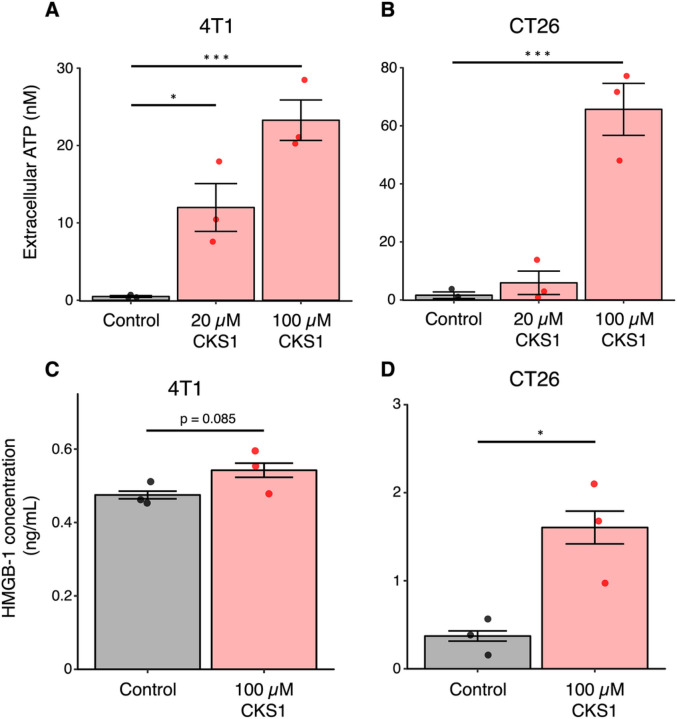
CKS1 induces release of immunogenic molecules. **(A, B**) Cells were treated with 20 μM or 100 μM CKS1 for 30 minutes and the amount of ATP released in the culture media was quantified. Means ± SEM, N = 3, Dunnet’s test (*p < 0.05, ***p < 0.005). (**C, D**) Cells were treated with 100 μM CKS1 for 6 hours and the amount of HMGB-1 released in the culture media was quantified. Means ± SEM, N = 3, Welch’s t-test (*p <0.05).

The release of damage-associated molecular patterns (DAMPs) such as ATP and HMGB-1 is a representative characteristic of immunogenic cell death [25]. To determine whether cell death caused by CKS1 is immunogenic, we evaluated the amount of ATP and HMGB-1 released in the culture media upon CKS1 treatment of 4T1 and CT26 cancer cells. During the process of immunogenic cell death, ATP is released before cell death, while HMGB-1 is released after cell death [26]. Therefore, we observed the release of ATP 30 minutes after CKS1 treatment, and the release of HMGB-1 6 hours after treatment. We observed a significant release of ATP from both cell lines (Fig. 6A, B). Although the release of HMGB-1 from 4T1 cells treated with CKS1 was weak, CT26 cells treated with CKS1 showed a significant release of HMGB-1 (Fig. 6C, D). These data indicate that CKS1 induces immunogenic cell death.

### CKS1 induces necrosis and inhibits tumor growth in vivo

**Figure 7:**
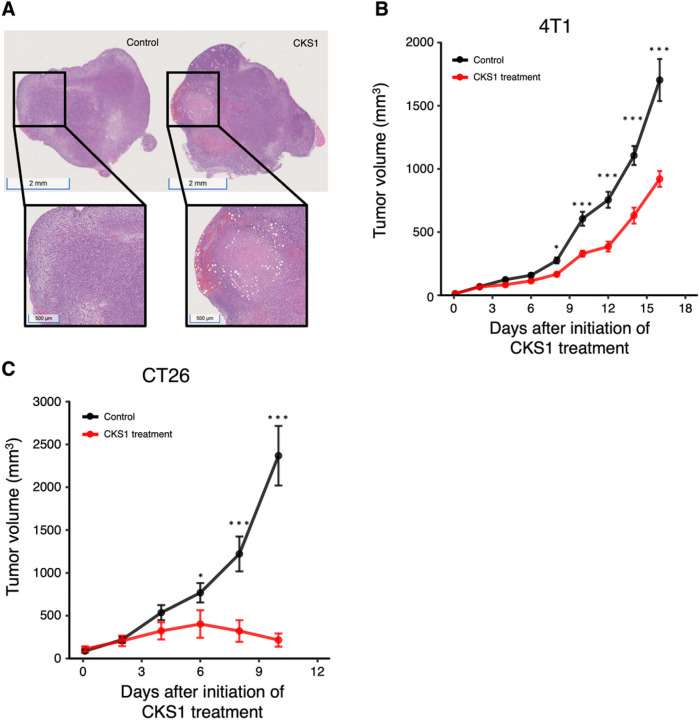
CKS1 induces necrosis and suppresses tumor growth in vivo. (**A**) CKS1 was injected intratumorally in established 4T1 tumors. After 24 hours, tumors were excised and were stained with H&E stain. The white area indicates necrosis caused by CKS1. Representative of N = 3. (**B, C**) Tumor volume of 4T1 tumors and CT26 tumors were recorded. 40 mg/kg CKS1 was applied daily starting on day 7. Means ± SEM, 4T1 control N = 15, 4T1 CKS1 treatment N =15, CT26 control N = 10, CT26 CKS1 treatment N = 10. Two-way repeated-measures ANOVA was performed to determine the significance of CKS1 treatment (***p < 0.005).

To evaluate whether CKS1 can induce cancer cell death in vivo, we injected CKS1 intratumorally into established 4T1 tumors. Intratumoral injection was chosen to ensure the delivery of peptides to the tumors. Necrosis was observed in tumors 24 hours after CKS1 treatment (Fig. 7A). Furthermore, the growth of both 4T1 tumors and CT26 tumors was inhibited by CKS1 treatment (Fig. 7B, 7C).

## Discussion

Our data demonstrate that CKS1 induces cell death in cancer cells with low cytotoxicity to healthy cells. We observed that the cell membrane of cancer cells is ruptured by CKS1 within 30 minutes of peptide treatment which indicates a necrotic cell death. On the other hand, we observed that cells show an apoptotic morphology at later time points after peptide treatment. These observations indicate that cancer cell death caused by CKS1 is a two-step process: first, the peptides accumulate on the cell surface of the cancer cells and create pores in the cell membrane, and second, the peptides enter the cells and activate the apoptotic pathway.

Although membrane rupture is a common phenomenon caused by oncolytic peptides, the induction of apoptosis is only seen in a few oncolytic peptides. For example, LTX-315 failed to stimulate caspase-3 activity, while inducing a necrotic cell death [27]. The factors that determine whether a peptide induces apoptosis or not are not known. Our data indicate that the mitochondrial membrane potential rapidly diminishes after peptide treatment. Although the mechanism of how CKS1 treatment affects the mitochondria remains unanswered, the malfunctioning of mitochondria caused by CKS1 may be the trigger of apoptotic cell death.

Our data showed that cells treated with CKS1 released immunogenic molecules. Anti-angiogenic agents improve the quality of the vasculature in the tumor (vascular normalization), leading to the alleviation of an immunosuppressive environment caused by hypoxia and acidosis and infiltration of immune effector cells [28]. Therefore, CKS1 may induce anti-cancer immunity by two means: release of immunogenic molecules and vascular normalization. The synergy between oncolytic peptides and cancer immunotherapy has been demonstrated for LTX-315 [23, 24, 29]. Our data show that CT26 tumors were highly sensitive to CKS1, while 4T1 tumors showed a relatively modest response to CKS1. This may be because CT26 cells are more immunogenic compared to 4T1 cells [26]. The potential of CKS1 as an immunotherapy has not been discussed in this study and further work would be required to determine the impact of CKS1 on anti-cancer immunity.

The obstacles to translating CKS1 to the clinic are the expected poor stability in vivo because of its allnatural amino acid composition and the need for a drug delivery system. To avoid these problems, we utilized intratumoral injection in the current study. Intratumoral injection can assure the accurate delivery of the drug to the tumor and can limit the risk of systemic side effects since it is a local administration method. Because of these benefits, intratumoral injection is adopted in multiple immunotherapies, such as immune receptor agonists and oncolytic viral therapies. Currently, more than 20 neoadjuvant clinical trials using these intratumoral immune stimulatory agents and their combinations are ongoing [30].

Intratumoral injection can only be applied to cancer types exposed on the surface of the body such as breast cancer and melanoma. Therefore, these cancer types may be good indications for treatment with CKS1.

In summary, CKS1 is a unique peptide that exerts anti-angiogenic and oncolytic activity to suppress tumor growth. Our experimental data indicate that cell membrane rupture and induction of the apoptotic pathway contribute to cell death. Since the oncolytic activity is not dependent on specific markers on cancer cells, CKS1 has the potential to be applied to a wide range of cancer types.

## Materials and Methods

### Peptides

CKS1 (NGRKACLNPASPIVKKIIEKMLNS), FITC-CKS1 (CKS1 with FITC tagged at the N-terminus), the helical region of CKS1 (IVKKIIEKMLNS), the loop region of CKS1 (NGRKACLNPASP), NC1 (NGRKACLNPASPIVEEIIEEMLNS), and NC2 (NGRKACLNPASPIVKKIIPEKMLNS) were synthesized by Genscript (Piscataway, NJ) using a solid-phase peptide synthesis method. There is a free amine at the Ntermini of all these peptides while they were all amidated at the C-terminus. The purity was > 90% as verified by HPLC and MS analyses. CKS1, the fragments of CKS1, and FITC-CKS1 were solubilized in water. NC1 and NC2 were solubilized in DMSO.

### Cell culture

4T1 murine mammary carcinoma cells (CRL-2539), CT26 murine colon carcinoma cells (CRL-2638), U2OS human osteosarcoma cells (HTB-96), and NIH/3T3 normal murine fibroblast cells (CRL-1658) were purchased from the American Type Culture Collection (Manassas, VA). MCA205 murine fibrosarcoma cells (SCC173) were purchased from MilliporeSigma (Burlington, MA). Cell lines were mycoplasma-tested before being used. 4T1 cells and MCA205 cells were propagated in RPMI 1640 (Corning, Corning, NY) supplemented with 10% FBS (MilliporeSigma). NIH/3T3 cells and U2OS cells were propagated in DMEM (Thermo Fisher Scientific, Waltham, MA) supplemented with 10% FBS. HUVECs were propagated in EGM-2 bulletkit (LONZA, Basel, Switzerland). All cell lines were grown in T75 tissue culture flasks (Sarstedt, Nümbrecht, Germany) under standard culture conditions of 37°C and 5% CO_2_.

### Prediction of the peptide structures

The structures of the peptides were predicted by the protein structure prediction software AlphaFold 2 [31]. The prediction of the protein structure was done using the ColabFold platform [32] and was visualized by Pymol [32]. The electrostatic property of the peptides was calculated using the APBS plugin in Pymol.

### Animal models

The protocols used in this study were approved by the Institutional Care and Use Committee at Johns Hopkins Medical Institutions. Four- to eight-week-old female Balb/c mice were obtained from Charles River (Wilmington, MA). For the 4T1 triple breast cancer model, 2.5 × 10^4^ 4T1 cells were injected into the first mammary fat pad of each mouse. For the CT26 colon carcinoma model, 1.0 × 10^6^ CT26 cells were injected subcutaneously in the left flank. After 1 week, we started daily intratumoral treatment with 40 mg/kg CKS1. The tumor size was measured by using a caliper, and the volume was calculated by using the formula 0.52 × (length) × (width)^2^.

### Real-time cell viability assay

3,000 cells were seeded in the wells of an xCELLigence RTCA E-plate 16 (Agilent, Santa Clara, CA). After the cells adhered to the plate, the media was replaced with serum free media, and the cells were treated with CKS1 at the indicated concentrations. The viability of the cells was monitored using the xCELLigence RTCA analyzer (Agilent).

### LDH assay

1.0 × 10^4^ cells were seeded in a 96-well plate and incubated overnight. The cells were washed once, and the medium was replaced with serum free media. Cells were treated with CKS1 for the indicated time and concentration. CyQUANT LDH cytotoxicity assay (Thermo Fisher Scientific) was used following the manufacturer’s instructions to measure the release of LDH. LDH release was normalized to the amount of LDH released from cells treated with lysis buffer.

### BrdU assay

1,000 cells were seeded in wells of a 96-well plate. The cells were treated with the indicated concentration of CKS1 immediately after seeding. 48 hours later, 5-bromo-2’-deoxyuridine (BrdU) was added to each well. 24 hours later, BrdU incorporation was quantified using the BrdU cell proliferation assay kit (Cell Signaling Technology, Danvers, MA) following the manufacturer’s instructions.

### Electron microscopy

Samples were fixed in 2.5% glutaraldehyde, 3 mM MgCl_2_ in 0.1 M sodium cacodylate buffer, pH 7.2 overnight at 4°C. After buffer rinse, samples were postfixed in 2% osmium tetroxide in 0.1 M sodium cacodylate for at least one hour (no more than two) on ice in the dark. After osmium, samples were rinsed in 0.1 M Maleate buffer (pH 6.2), followed by uranyl acetate in 0.1 M Maleate (0.22 μm filtered, 1 h, dark), dehydrated in a graded series of ethanol and embedded in Epon (PolySci) resin. Samples were polymerized at 60°C overnight. Thin sections, 60 to 90 nm, were cut with a diamond knife on a Leica UCT ultramicrotome and picked up with 2×1 mm Formvar copper slot grids. Grids were stained with 2% uranyl acetate (aq.) followed by lead citrate and observed with a Hitachi 7600 TEM at 80 kV. Images were captured with an AMT CCD XR80 (8-megapixel camera - side mount AMT XR80 – high-resolution highspeed camera).

### Fluorescence labeling

Fluorescence labeling of cells was performed by seeding 5.0 × 10^4^ 4T1 cells in a 35mm dish on a collagen-coated No. 1.5 coverslip with a 14 mm glass diameter (MatTek, Ashland, MA). After overnight incubation, the cells were washed once with serum free media and replaced with fresh serum free media. For cell membrane labeling, NR12S (R&D Systems, Minneapolis, MN, #7509) was added at a final concentration of 0.3 μM and incubated for 30 minutes. For cell cytosol labeling, Calcein-AM (Thermo Fisher Scientific, C1430) was added at a final concentration of 5 μg/mL and incubated for 10 minutes. For mitochondrial membrane labeling, JC-1 (Thermo Fisher Scientific, T3168) was added at a final concentration of 10 μg/mL and incubated for 10 minutes. For nuclear staining, Hoechst 33342 (Cell Signaling Technology, #4082) was added at a final concentration of 5 μg/mL and incubated for 10 minutes.

### Fluorescence microscopy

Cells were imaged on either a Zeiss LSM 700 or a 3i spinning disk confocal microscope. Samples were imaged with optimized microscope settings. Z-stack, time-lapse, or multi-channel acquisition settings were adjusted based on the experimental requirements. Images were then acquired with the appropriate excitation and emission wavelengths for each fluorophore.

### Image analysis

Image analysis was performed using Fiji software. For each image/video, the brightness and the contrast were adjusted. The time stamp and the scale bar were added.

### Caspase-3 activity assay

1.0 × 10^4^ 4T1 cells were seeded in 96-well plates and incubated overnight. After CKS1 treatment, the cells were lysed using lysis buffer (Cell Signaling Technology, #7018). The activity of caspase-3 in the cell lysate was quantified using the caspase-3 activity assay kit (Cell Signaling Technology, #5723) following the manufacturer’s instructions. Briefly, the cell lysate was incubated with a substrate of caspase-3 which was designed to emit fluorescence when cleaved. After incubation, the fluorescence was measured using a plate reader. The protein concentration of the cell lysate was quantified using the DC protein assay kit (Bio-Rad, Hercules, CA). The caspase-3 activity was divided by the protein concentration to obtain the caspase-3 activity per μg/mL protein in the cell lysate.

### Quantification of ATP release

1.5 × 10^4^ cells of 4T1 cells or CT26 cells in growth media were seeded in wells of a 96-well plate. After 12 hours, the media was replaced with serum free media, and the cells were treated with the corresponding concentration of CKS1. The cells were treated with CKS1 for 6 hours and the supernatant was collected from each well. After filtering the supernatant with a 0.2 μm pore filter (Corning, 431229), the amount of ATP in the supernatant was quantified using the ENLITEN ATP assay system (Promega Corporation, Madison, WI, FF2000). We followed the instructions from the manufacturer when using the ENLITEN ATP assay system.

### Quantification of HMGB-1 release

1.5 ×10^4^ cells of 4T1 cells or CT26 cells in growth media were seeded in wells of a 96-well plate. After 12 hours, the media was replaced with serum free media, and the cells were treated with the corresponding concentration of CKS1. The cells were treated with CKS1 for 6 hours and the supernatant was collected from each well. After filtering the supernatant with a 0.2 μm pore filter (Corning, 431229), the amount of HMGB-1 in the supernatant was quantified using the Mouse/Rat HMGB1 ELISA kit (arigo biolaboratories, ARG81310).

### H&E staining

Tumors were fixed by immersion in 10% neutral buffered formalin (Millipore Sigma) for 48 hours and subsequently dehydrated by immersion in 70%, 90%, and 100% ethanol followed by xylene for two changes of 30 minutes each. Tissues were embedded in paraffin blocks at 58°C and sectioned at 4 μm using a microtome. Sections were floated in a water bath at 56°C, embedded onto glass slides, and dried overnight. Next, sections were rehydrated by immersion in xylene, followed by 100%, 95%, 70% ethanol, and finally water for two changes of 5 minutes each. For staining, slides were immersed in Gill’s Hematoxylin #2 (Millipore Sigma) for 1 minute, immediately rinsed in tap water to prevent overstaining, and immersed in Eosin Y (Millipore Sigma) for 1 minute and rinsed. Finally, slides were again dehydrated, mounted with Citramount, coverslipped, and scanned at 40X with a NanoZoomer slide scanner.

### Statistics

All statistics analyses were performed using R. Differences between two groups were determined using unpaired two-tailed Welch’s t-test and were considered statistically significant when p < 0.05. To determine the difference between the control group and the treatment groups, we used Dunnett’s test. We used twoway repeated-measures ANOVA to determine the significance of the treatment in the in vivo experiment monitoring tumor growth. All data were replicated in at least three independent experiments and the results are expressed as mean ± SEM.
